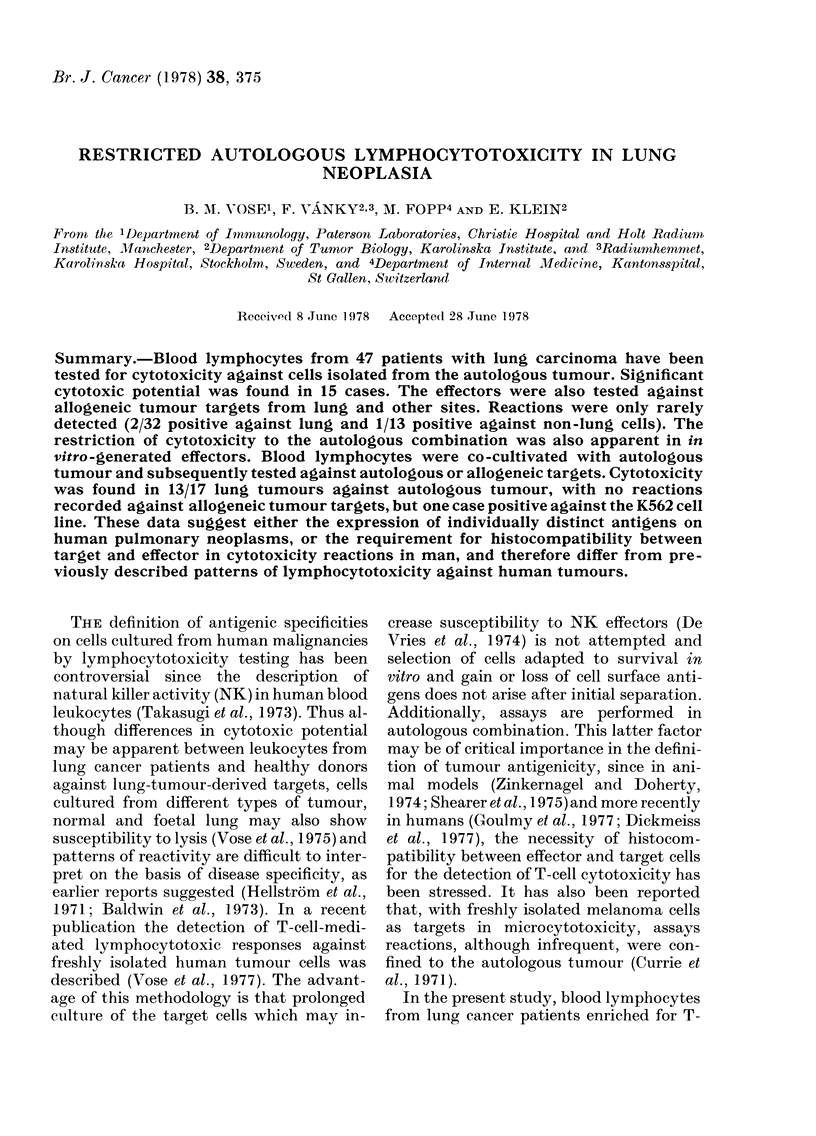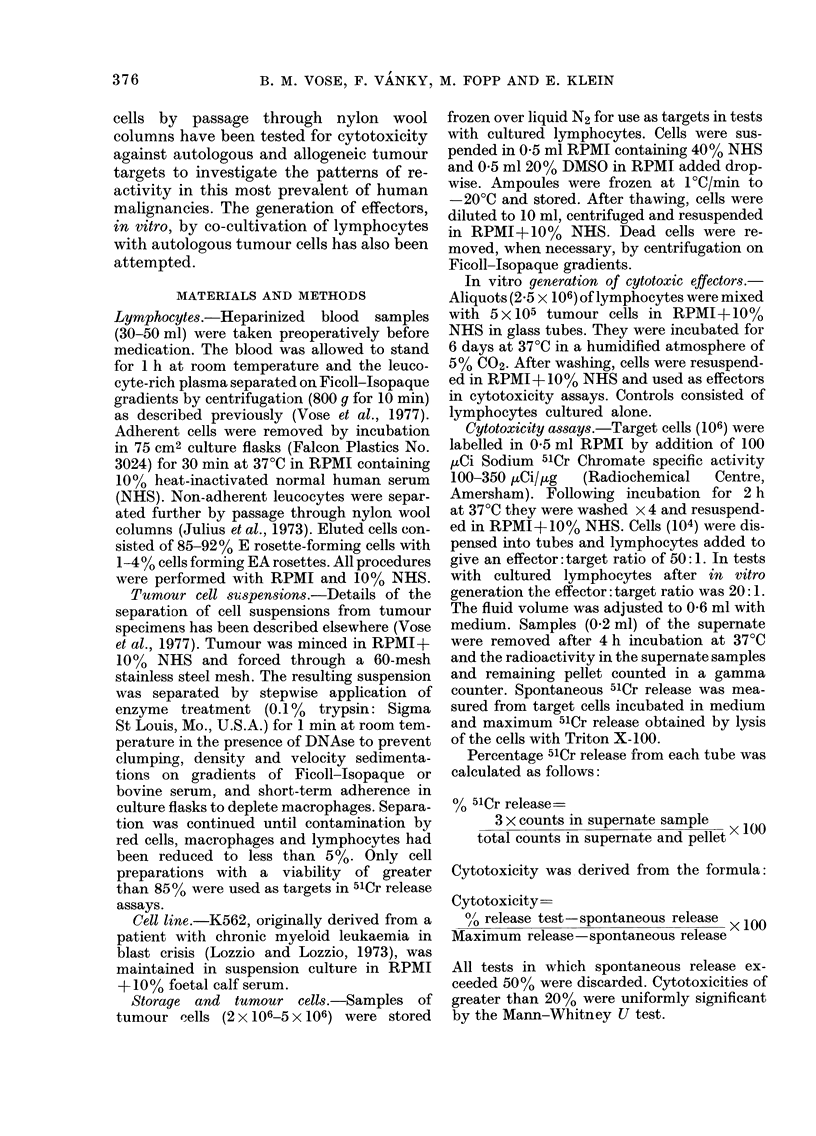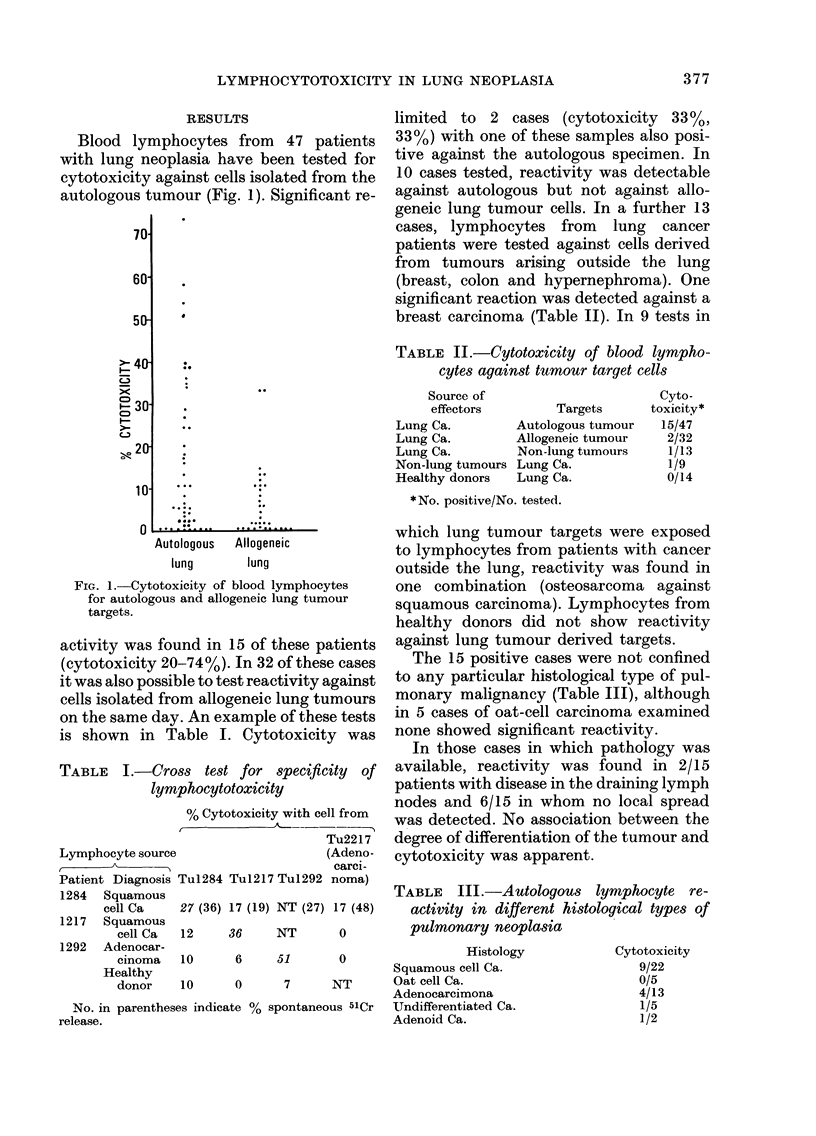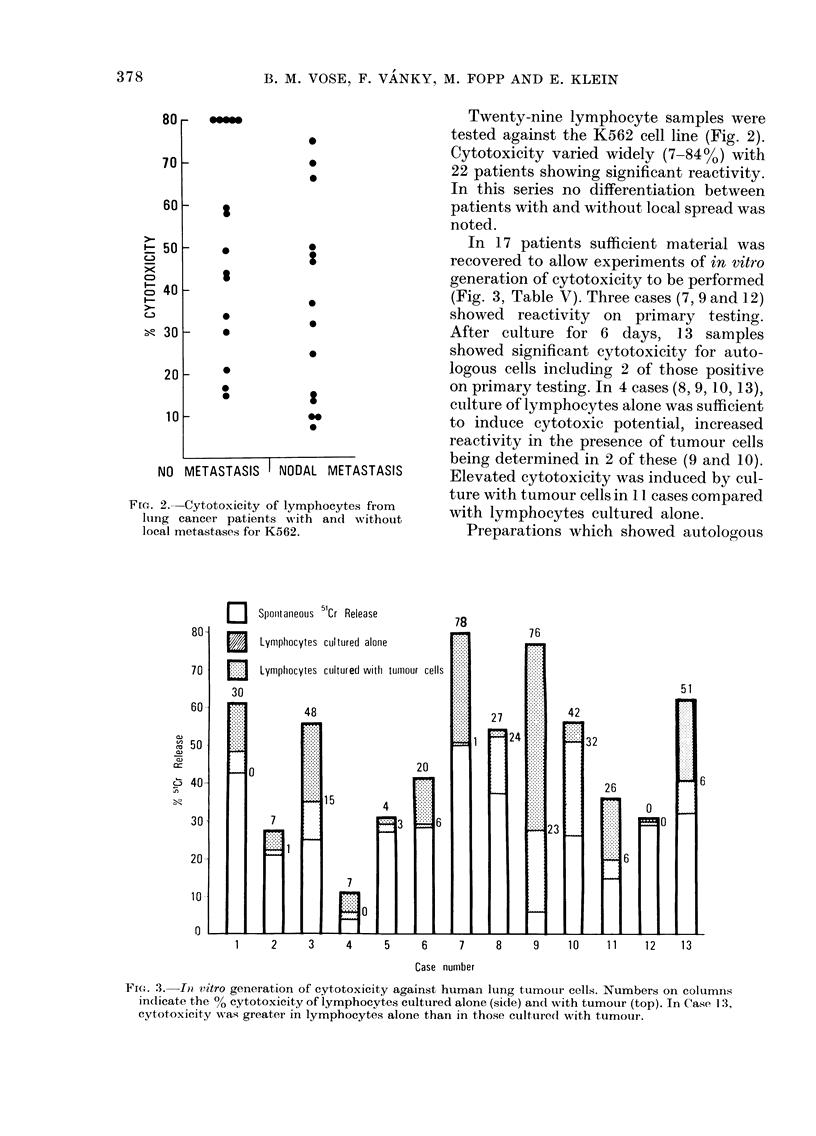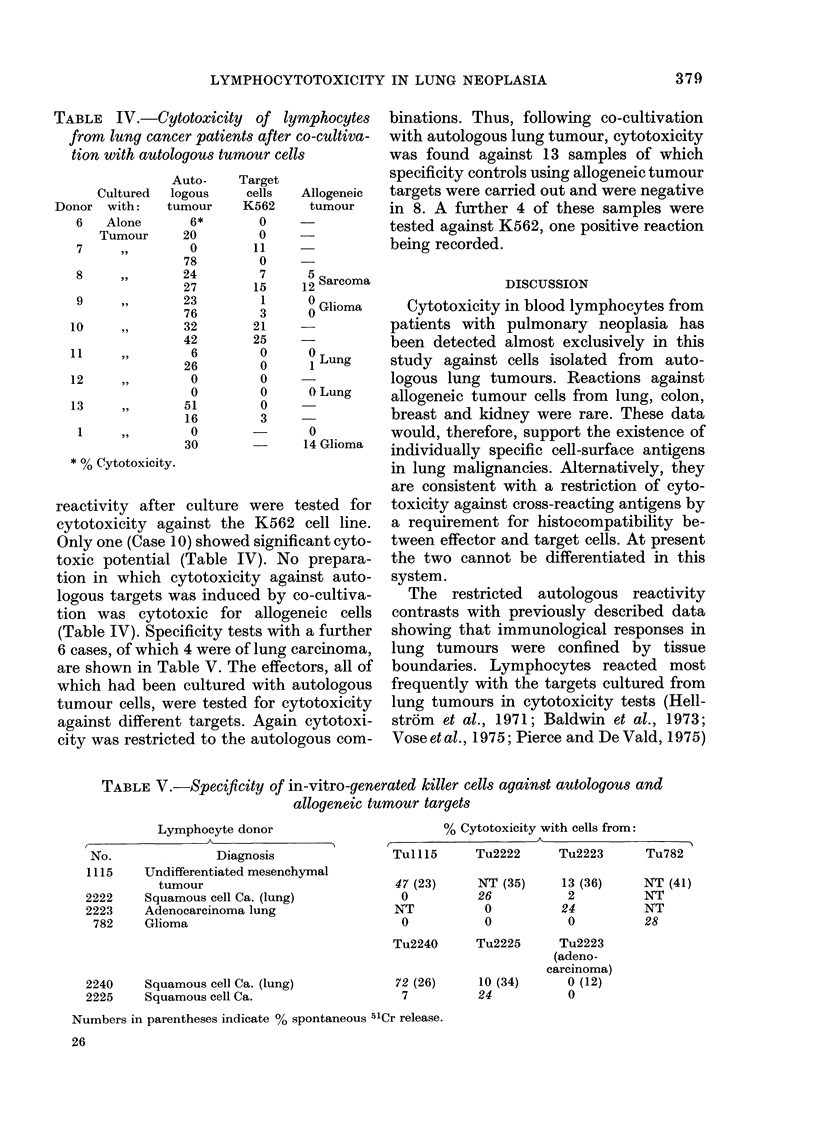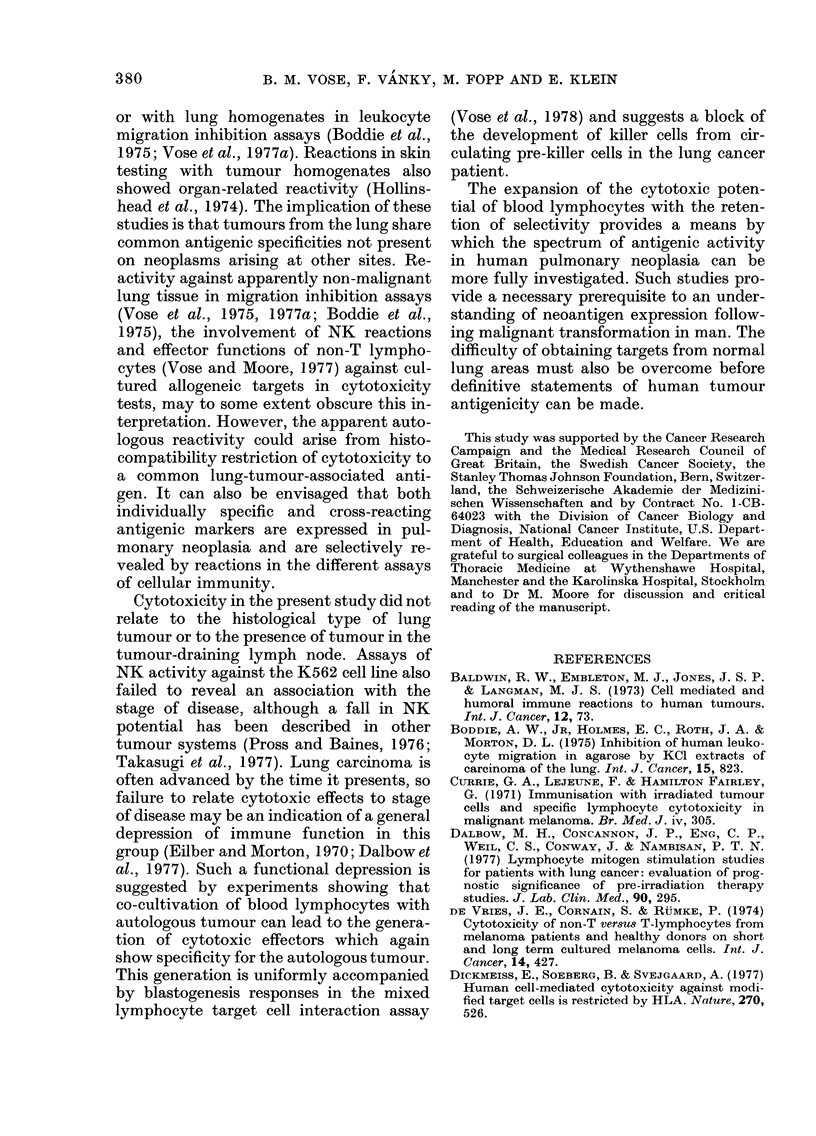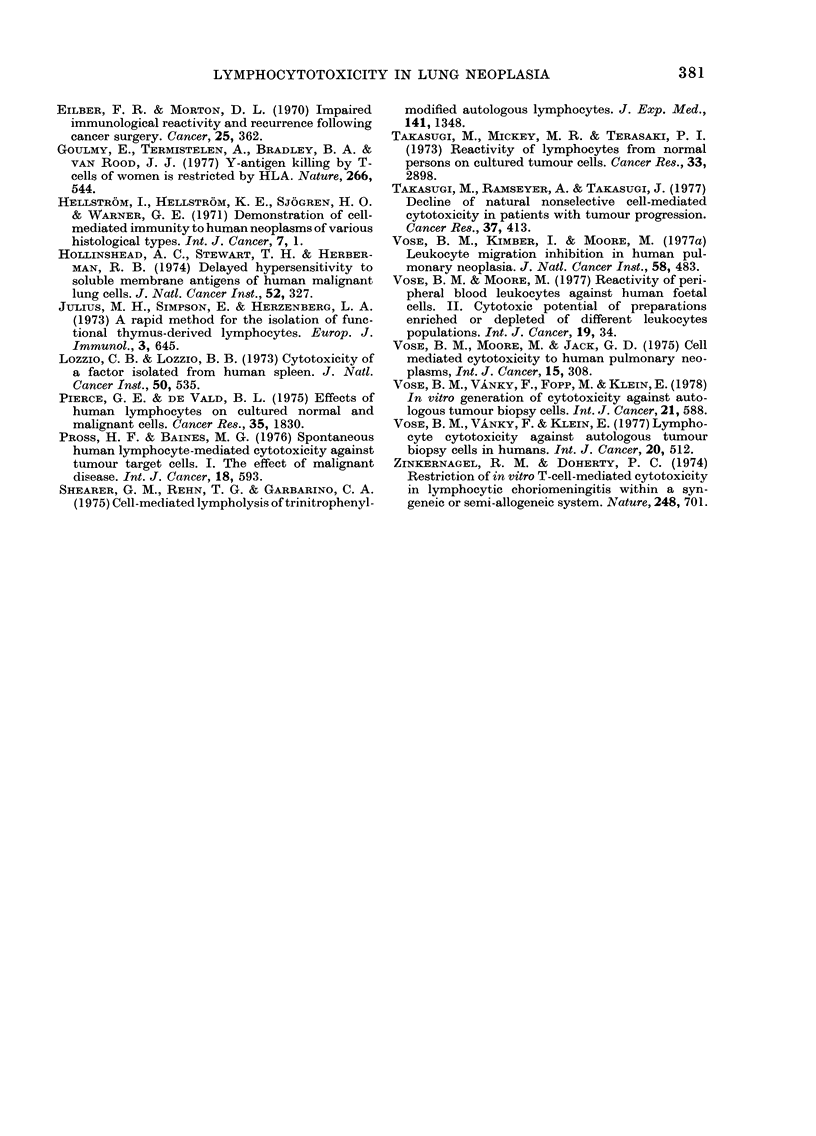# Restricted autologous lymphocytotoxicity in lung neoplasia.

**DOI:** 10.1038/bjc.1978.217

**Published:** 1978-09

**Authors:** B. M. Vose, F. Vánky, M. Fopp, E. Klein

## Abstract

Blood lymphocytes from 47 patients with lung carcinoma have been tested for cytotoxicity against cells isolated from the autologous tumour. Significant cytotoxic potential was found in 15 cases. The effectors were also tested against allogeneic tumour targets from lung and other sites. Reactions were only rarely detected (2/32 positive against lung and 1/13 positive against non-lung cells). The restriction of cytotoxicity to the autologous combination was also apparent in in vitro-generated effectors. Blood lymphocytes were co-cultivated with autologous tumour and subsequently tested against autologous or allogeneic targets. Cytotoxicity was found in 13/17 lung tumours against autologous tumour, with no reactions recorded against allogeneic tumour targets, but one case positive against the K562 cell line. These data suggest either the expression of individually distinct antigens on human pulmonary neoplasms, or the requirement for histocompatibility between target and effector in cytotoxicity reactions in man, and therefore differ from previously described patterns of lymphocytotoxicity against human tumours.


*Br. J. Cancer* (1978) **38**, 375

### RESTRICTED AUTOLOGOUS LYMPHOCYTOTOXICITY IN LUNG NEOPLASIA

B. M. VOSE[1], F. VÁNKY[2,3], M. FOPP[4] AND E. KLEIN[2]

*From the [1]Department of Immunology, Paterson Laboratories, Christie Hospital and Holt Radium Institute, Manchester, [2]Department of Tumor Biology, Karolinska Institute, and [3]Radiumhemmet, Karolinska Hospital, Stockholm, Sweden, and [4]Department of Internal Medicine, Kantonsspital, St Gallen, Switzerland*

Received 8 June 1978 Accepted 28 June 1978

**Summary.—Blood lymphocytes from 47 patients with lung carcinoma have been tested for cytotoxicity against cells isolated from the autologous tumour. Significant cytotoxic potential was found in 15 cases. The effectors were also tested against allogeneic tumour targets from lung and other sites. Reactions were only rarely detected (2/32 positive against lung and 1/13 positive against non-lung cells). The restriction of cytotoxicity to the autologous combination was also apparent in *in vitro*-generated effectors. Blood lymphocytes were co-cultivated with autologous tumour and subsequently tested against autologous or allogeneic targets. Cytotoxicity was found in 13/17 lung tumours against autologous tumour, with no reactions recorded against allogeneic tumour targets, but one case positive against the K562 cell line. These data suggest either the expression of individually distinct antigens on human pulmonary neoplasms, or the requirement for histocompatibility between target and effector in cytotoxicity reactions in man, and therefore differ from previously described patterns of lymphocytotoxicity against human tumours.**

THE definition of antigenic specificities on cells cultured from human malignancies by lymphocytotoxicity testing has been controversial since the description of natural killer activity (NK) in human blood leukocytes (Takasugi *et al.*, 1973). Thus although differences in cytotoxic potential may be apparent between leukocytes from lung cancer patients and healthy donors against lung-tumour-derived targets, cells cultured from different types of tumour, normal and foetal lung may also show susceptibility to lysis (Vose *et al.*, 1975) and patterns of reactivity are difficult to interpret on the basis of disease specificity, as earlier reports suggested (Hellström *et al.*, 1971; Baldwin *et al.*, 1973). In a recent publication the detection of T-cell-mediated lymphocytotoxic responses against freshly isolated human tumour cells was described (Vose *et al.*, 1977). The advantage of this methodology is that prolonged culture of the target cells which may increase susceptibility to NK effectors (De Vries *et al.*, 1974) is not attempted and selection of cells adapted to survival *in vitro* and gain or loss of cell surface antigens does not arise after initial separation. Additionally, assays are performed in autologous combination. This latter factor may be of critical importance in the definition of tumour antigenicity, since in animal models (Zinkernagel and Doherty, 1974; Shearer *et al.*, 1975) and more recently in humans (Goulmy *et al.*, 1977; Dickmeiss *et al.*, 1977), the necessity of histocompatibility between effector and target cells for the detection of T-cell cytotoxicity has been stressed. It has also been reported that, with freshly isolated melanoma cells as targets in microcytotoxicity, assays reactions, although infrequent, were confined to the autologous tumour (Currie *et al.*, 1971).

In the present study, blood lymphocytes from lung cancer patients enriched for T-

cells by passage through nylon wool columns have been tested for cytotoxicity against autologous and allogeneic tumour targets to investigate the patterns of reactivity in this most prevalent of human malignancies. The generation of effectors, *in vitro*, by co-cultivation of lymphocytes with autologous tumour cells has also been attempted.

### MATERIALS AND METHODS

*Lymphocytes.*—Heparinized blood samples (30–50 ml) were taken preoperatively before medication. The blood was allowed to stand for 1 h at room temperature and the leucocyte-rich plasma separated on Ficoll–Isopaque gradients by centrifugation (800 *g* for 10 min) as described previously (Vose *et al.*, 1977). Adherent cells were removed by incubation in 75 $cm^2$ culture flasks (Falcon Plastics No. 3024) for 30 min at 37°C in RPMI containing 10% heat-inactivated normal human serum (NHS). Non-adherent leucocytes were separated further by passage through nylon wool columns (Julius *et al.*, 1973). Eluted cells consisted of 85–92% E rosette-forming cells with 1–4% cells forming EA rosettes. All procedures were performed with RPMI and 10% NHS.

*Tumour cell suspensions.*—Details of the separation of cell suspensions from tumour specimens has been described elsewhere (Vose *et al.*, 1977). Tumour was minced in RPMI+10% NHS and forced through a 60-mesh stainless steel mesh. The resulting suspension was separated by stepwise application of enzyme treatment (0.1% trypsin: Sigma St Louis, Mo., U.S.A.) for 1 min at room temperature in the presence of DNAse to prevent clumping, density and velocity sedimentations on gradients of Ficoll–Isopaque or bovine serum, and short-term adherence in culture flasks to deplete macrophages. Separation was continued until contamination by red cells, macrophages and lymphocytes had been reduced to less than 5%. Only cell preparations with a viability of greater than 85% were used as targets in $^{51}$Cr release assays.

*Cell line.*—K562, originally derived from a patient with chronic myeloid leukaemia in blast crisis (Lozzio and Lozzio, 1973), was maintained in suspension culture in RPMI +10% foetal calf serum.

*Storage and tumour cells.*—Samples of tumour cells ($2\times10^6$–$5\times10^6$) were stored frozen over liquid $N_2$ for use as targets in tests with cultured lymphocytes. Cells were suspended in 0·5 ml RPMI containing 40% NHS and 0·5 ml 20% DMSO in RPMI added dropwise. Ampoules were frozen at 1°C/min to −20°C and stored. After thawing, cells were diluted to 10 ml, centrifuged and resuspended in RPMI+10% NHS. Dead cells were removed, when necessary, by centrifugation on Ficoll–Isopaque gradients.

In vitro *generation of cytotoxic effectors.*—Aliquots ($2{\cdot}5\times10^6$) of lymphocytes were mixed with $5\times10^5$ tumour cells in RPMI+10% NHS in glass tubes. They were incubated for 6 days at 37°C in a humidified atmosphere of 5% $CO_2$. After washing, cells were resuspended in RPMI+10% NHS and used as effectors in cytotoxicity assays. Controls consisted of lymphocytes cultured alone.

*Cytotoxicity assays.*—Target cells ($10^6$) were labelled in 0·5 ml RPMI by addition of 100 μCi Sodium $^{51}$Cr Chromate specific activity 100–350 μCi/μg (Radiochemical Centre, Amersham). Following incubation for 2 h at 37°C they were washed ×4 and resuspended in RPMI+10% NHS. Cells ($10^4$) were dispensed into tubes and lymphocytes added to give an effector:target ratio of 50:1. In tests with cultured lymphocytes after *in vitro* generation the effector:target ratio was 20:1. The fluid volume was adjusted to 0·6 ml with medium. Samples (0·2 ml) of the supernate were removed after 4 h incubation at 37°C and the radioactivity in the supernate samples and remaining pellet counted in a gamma counter. Spontaneous $^{51}$Cr release was measured from target cells incubated in medium and maximum $^{51}$Cr release obtained by lysis of the cells with Triton X-100.

Percentage $^{51}$Cr release from each tube was calculated as follows:

$$\% \; ^{51}\text{Cr release} = \frac{3\times\text{counts in supernate sample}}{\text{total counts in supernate and pellet}}\times100$$

Cytotoxicity was derived from the formula:

$$\text{Cytotoxicity} = \frac{\%\ \text{release test} - \text{spontaneous release}}{\text{Maximum release} - \text{spontaneous release}}\times100$$

All tests in which spontaneous release exceeded 50% were discarded. Cytotoxicities of greater than 20% were uniformly significant by the Mann–Whitney *U* test.

### RESULTS

Blood lymphocytes from 47 patients with lung neoplasia have been tested for cytotoxicity against cells isolated from the autologous tumour (Fig. 1). Significant re-

FIG. 1.—Cytotoxicity of blood lymphocytes for autologous and allogeneic lung tumour targets.

activity was found in 15 of these patients (cytotoxicity 20–74%). In 32 of these cases it was also possible to test reactivity against cells isolated from allogeneic lung tumours on the same day. An example of these tests is shown in Table I. Cytotoxicity was limited to 2 cases (cytotoxicity 33%, 33%) with one of these samples also positive against the autologous specimen. In 10 cases tested, reactivity was detectable against autologous but not against allogeneic lung tumour cells. In a further 13 cases, lymphocytes from lung cancer patients were tested against cells derived from tumours arising outside the lung (breast, colon and hypernephroma). One significant reaction was detected against a breast carcinoma (Table II). In 9 tests in which lung tumour targets were exposed to lymphocytes from patients with cancer outside the lung, reactivity was found in one combination (osteosarcoma against squamous carcinoma). Lymphocytes from healthy donors did not show reactivity against lung tumour derived targets.

TABLE I.—*Cross test for specificity of lymphocytotoxicity*

| Lymphocyte source | | % Cytotoxicity with cell from | | | |
|---|---|---|---|---|---|
| Patient | Diagnosis | Tu1284 | Tu1217 | Tu1292 | Tu2217 (Adenocarcinoma) |
| 1284 | Squamous cell Ca | *27* (36) | 17 (19) | NT (27) | 17 (48) |
| 1217 | Squamous cell Ca | 12 | *36* | NT | 0 |
| 1292 | Adenocarcinoma | 10 | 6 | *51* | 0 |
| | Healthy donor | 10 | 0 | 7 | NT |

No. in parentheses indicate % spontaneous $^{51}Cr$ release.

TABLE II.—*Cytotoxicity of blood lymphocytes against tumour target cells*

| Source of effectors | Targets | Cytotoxicity* |
|---|---|---|
| Lung Ca. | Autologous tumour | 15/47 |
| Lung Ca. | Allogeneic tumour | 2/32 |
| Lung Ca. | Non-lung tumours | 1/13 |
| Non-lung tumours | Lung Ca. | 1/9 |
| Healthy donors | Lung Ca. | 0/14 |

*No. positive/No. tested.

The 15 positive cases were not confined to any particular histological type of pulmonary malignancy (Table III), although in 5 cases of oat-cell carcinoma examined none showed significant reactivity.

In those cases in which pathology was available, reactivity was found in 2/15 patients with disease in the draining lymph nodes and 6/15 in whom no local spread was detected. No association between the degree of differentiation of the tumour and cytotoxicity was apparent.

TABLE III.—*Autologous lymphocyte reactivity in different histological types of pulmonary neoplasia*

| Histology | Cytotoxicity |
|---|---|
| Squamous cell Ca. | 9/22 |
| Oat cell Ca. | 0/5 |
| Adenocarcimona | 4/13 |
| Undifferentiated Ca. | 1/5 |
| Adenoid Ca. | 1/2 |

FIG. 2.—Cytotoxicity of lymphocytes from lung cancer patients with and without local metastases for K562.

Twenty-nine lymphocyte samples were tested against the K562 cell line (Fig. 2). Cytotoxicity varied widely (7–84%) with 22 patients showing significant reactivity. In this series no differentiation between patients with and without local spread was noted.

In 17 patients sufficient material was recovered to allow experiments of *in vitro* generation of cytotoxicity to be performed (Fig. 3, Table V). Three cases (7, 9 and 12) showed reactivity on primary testing. After culture for 6 days, 13 samples showed significant cytotoxicity for autologous cells including 2 of those positive on primary testing. In 4 cases (8, 9, 10, 13), culture of lymphocytes alone was sufficient to induce cytotoxic potential, increased reactivity in the presence of tumour cells being determined in 2 of these (9 and 10). Elevated cytotoxicity was induced by culture with tumour cells in 11 cases compared with lymphocytes cultured alone.

Preparations which showed autologous

FIG. 3.—*In vitro* generation of cytotoxicity against human lung tumour cells. Numbers on columns indicate the % cytotoxicity of lymphocytes cultured alone (side) and with tumour (top). In Case 13, cytotoxicity was greater in lymphocytes alone than in those cultured with tumour.

TABLE IV.—*Cytotoxicity of lymphocytes from lung cancer patients after co-cultivation with autologous tumour cells*

| Donor | Cultured with: | Autologous tumour | Target cells K562 | Allogeneic tumour |
|---|---|---|---|---|
| 6 | Alone | 6* | 0 | — |
| | Tumour | 20 | 0 | — |
| 7 | ,, | 0 | 11 | — |
| | | 78 | 0 | — |
| 8 | ,, | 24 | 7 | 5 Sarcoma |
| | | 27 | 15 | 12 Sarcoma |
| 9 | ,, | 23 | 1 | 0 Glioma |
| | | 76 | 3 | 0 Glioma |
| 10 | ,, | 32 | 21 | — |
| | | 42 | 25 | — |
| 11 | ,, | 6 | 0 | 0 Lung |
| | | 26 | 0 | 1 Lung |
| 12 | ,, | 0 | 0 | — |
| | | 0 | 0 | 0 Lung |
| 13 | ,, | 51 | 0 | — |
| | | 16 | 3 | — |
| 1 | ,, | 0 | — | 0 |
| | | 30 | — | 14 Glioma |

* % Cytotoxicity.

reactivity after culture were tested for cytotoxicity against the K562 cell line. Only one (Case 10) showed significant cytotoxic potential (Table IV). No preparation in which cytotoxicity against autologous targets was induced by co-cultivation was cytotoxic for allogeneic cells (Table IV). Specificity tests with a further 6 cases, of which 4 were of lung carcinoma, are shown in Table V. The effectors, all of which had been cultured with autologous tumour cells, were tested for cytotoxicity against different targets. Again cytotoxicity was restricted to the autologous combinations. Thus, following co-cultivation with autologous lung tumour, cytotoxicity was found against 13 samples of which specificity controls using allogeneic tumour targets were carried out and were negative in 8. A further 4 of these samples were tested against K562, one positive reaction being recorded.

### DISCUSSION

Cytotoxicity in blood lymphocytes from patients with pulmonary neoplasia has been detected almost exclusively in this study against cells isolated from autologous lung tumours. Reactions against allogeneic tumour cells from lung, colon, breast and kidney were rare. These data would, therefore, support the existence of individually specific cell-surface antigens in lung malignancies. Alternatively, they are consistent with a restriction of cytotoxicity against cross-reacting antigens by a requirement for histocompatibility between effector and target cells. At present the two cannot be differentiated in this system.

The restricted autologous reactivity contrasts with previously described data showing that immunological responses in lung tumours were confined by tissue boundaries. Lymphocytes reacted most frequently with the targets cultured from lung tumours in cytotoxicity tests (Hellström *et al.*, 1971; Baldwin *et al.*, 1973; Vose *et al.*, 1975; Pierce and De Vald, 1975)

TABLE V.—*Specificity of* in-vitro-*generated killer cells against autologous and allogeneic tumour targets*

| Lymphocyte donor | | % Cytotoxicity with cells from: | | | |
|---|---|---|---|---|---|
| No. | Diagnosis | Tu1115 | Tu2222 | Tu2223 | Tu782 |
| 1115 | Undifferentiated mesenchymal tumour | *47* (23) | NT (35) | 13 (36) | NT (41) |
| 2222 | Squamous cell Ca. (lung) | 0 | *26* | 2 | NT |
| 2223 | Adenocarcinoma lung | NT | 0 | *24* | NT |
| 782 | Glioma | 0 | 0 | 0 | *28* |
| | | Tu2240 | Tu2225 | Tu2223 (adenocarcinoma) | |
| 2240 | Squamous cell Ca. (lung) | *72* (26) | 10 (34) | 0 (12) | |
| 2225 | Squamous cell Ca. | 7 | *24* | 0 | |

Numbers in parentheses indicate % spontaneous $^{51}Cr$ release.

or with lung homogenates in leukocyte migration inhibition assays (Boddie *et al.*, 1975; Vose *et al.*, 1977*a*). Reactions in skin testing with tumour homogenates also showed organ-related reactivity (Hollinshead *et al.*, 1974). The implication of these studies is that tumours from the lung share common antigenic specificities not present on neoplasms arising at other sites. Reactivity against apparently non-malignant lung tissue in migration inhibition assays (Vose *et al.*, 1975, 1977*a*; Boddie *et al.*, 1975), the involvement of NK reactions and effector functions of non-T lymphocytes (Vose and Moore, 1977) against cultured allogeneic targets in cytotoxicity tests, may to some extent obscure this interpretation. However, the apparent autologous reactivity could arise from histocompatibility restriction of cytotoxicity to a common lung-tumour-associated antigen. It can also be envisaged that both individually specific and cross-reacting antigenic markers are expressed in pulmonary neoplasia and are selectively revealed by reactions in the different assays of cellular immunity.

Cytotoxicity in the present study did not relate to the histological type of lung tumour or to the presence of tumour in the tumour-draining lymph node. Assays of NK activity against the K562 cell line also failed to reveal an association with the stage of disease, although a fall in NK potential has been described in other tumour systems (Pross and Baines, 1976; Takasugi *et al.*, 1977). Lung carcinoma is often advanced by the time it presents, so failure to relate cytotoxic effects to stage of disease may be an indication of a general depression of immune function in this group (Eilber and Morton, 1970; Dalbow *et al.*, 1977). Such a functional depression is suggested by experiments showing that co-cultivation of blood lymphocytes with autologous tumour can lead to the generation of cytotoxic effectors which again show specificity for the autologous tumour. This generation is uniformly accompanied by blastogenesis responses in the mixed lymphocyte target cell interaction assay (Vose *et al.*, 1978) and suggests a block of the development of killer cells from circulating pre-killer cells in the lung cancer patient.

The expansion of the cytotoxic potential of blood lymphocytes with the retention of selectivity provides a means by which the spectrum of antigenic activity in human pulmonary neoplasia can be more fully investigated. Such studies provide a necessary prerequisite to an understanding of neoantigen expression following malignant transformation in man. The difficulty of obtaining targets from normal lung areas must also be overcome before definitive statements of human tumour antigenicity can be made.

This study was supported by the Cancer Research Campaign and the Medical Research Council of Great Britain, the Swedish Cancer Society, the Stanley Thomas Johnson Foundation, Bern, Switzerland, the Schweizerische Akademie der Medizinischen Wissenschaften and by Contract No. 1-CB-64023 with the Division of Cancer Biology and Diagnosis, National Cancer Institute, U.S. Department of Health, Education and Welfare. We are grateful to surgical colleagues in the Departments of Thoracic Medicine at Wythenshawe Hospital, Manchester and the Karolinska Hospital, Stockholm and to Dr M. Moore for discussion and critical reading of the manuscript.

### REFERENCES

Baldwin, R. W., Embleton, M. J., Jones, J. S. P. & Langman, M. J. S. (1973) Cell mediated and humoral immune reactions to human tumours. *Int. J. Cancer*, **12**, 73.

Boddie, A. W., Jr, Holmes, E. C., Roth, J. A. & Morton, D. L. (1975) Inhibition of human leukocyte migration in agarose by KCl extracts of carcinoma of the lung. *Int. J. Cancer*, **15**, 823.

Currie, G. A., Lejeune, F. & Hamilton Fairley, G. (1971) Immunisation with irradiated tumour cells and specific lymphocyte cytotoxicity in malignant melanoma. *Br. Med. J.* iv, 305.

Dalbow, M. H., Concannon, J. P., Eng, C. P., Weil, C. S., Conway, J. & Nambisan, P. T. N. (1977) Lymphocyte mitogen stimulation studies for patients with lung cancer: evaluation of prognostic significance of pre-irradiation therapy studies. *J. Lab. Clin. Med.*, **90**, 295.

de Vries, J. E., Cornain, S. & Rümke, P. (1974) Cytotoxicity of non-T *versus* T-lymphocytes from melanoma patients and healthy donors on short and long term cultured melanoma cells. *Int. J. Cancer*, **14**, 427.

Dickmeiss, E., Soeberg, B. & Svejgaard, A. (1977) Human cell-mediated cytotoxicity against modified target cells is restricted by HLA. *Nature*, **270**, 526.

EILBER, F. R. & MORTON, D. L. (1970) Impaired immunological reactivity and recurrence following cancer surgery. *Cancer*, **25**, 362.

GOULMY, E., TERMISTELEN, A., BRADLEY, B. A. & VAN ROOD, J. J. (1977) Y-antigen killing by T-cells of women is restricted by HLA. *Nature*, **266**, 544.

HELLSTRÖM, I., HELLSTRÖM, K. E., SJÖGREN, H. O. & WARNER, G. E. (1971) Demonstration of cell-mediated immunity to human neoplasms of various histological types. *Int. J. Cancer*, **7**, 1.

HOLLINSHEAD, A. C., STEWART, T. H. & HERBERMAN, R. B. (1974) Delayed hypersensitivity to soluble membrane antigens of human malignant lung cells. *J. Natl. Cancer Inst.*, **52**, 327.

JULIUS, M. H., SIMPSON, E. & HERZENBERG, L. A. (1973) A rapid method for the isolation of functional thymus-derived lymphocytes. *Europ. J. Immunol.*, **3**, 645.

LOZZIO, C. B. & LOZZIO, B. B. (1973) Cytotoxicity of a factor isolated from human spleen. *J. Natl. Cancer Inst.*, **50**, 535.

PIERCE, G. E. & DE VALD, B. L. (1975) Effects of human lymphocytes on cultured normal and malignant cells. *Cancer Res.*, **35**, 1830.

PROSS, H. F. & BAINES, M. G. (1976) Spontaneous human lymphocyte-mediated cytotoxicity against tumour target cells. I. The effect of malignant disease. *Int. J. Cancer*, **18**, 593.

SHEARER, G. M., REHN, T. G. & GARBARINO, C. A. (1975) Cell-mediated lympholysis of trinitrophenyl-modified autologous lymphocytes. *J. Exp. Med.*, **141**, 1348.

TAKASUGI, M., MICKEY, M. R. & TERASAKI, P. I. (1973) Reactivity of lymphocytes from normal persons on cultured tumour cells. *Cancer Res.*, **33**, 2898.

TAKASUGI, M., RAMSEYER, A. & TAKASUGI, J. (1977) Decline of natural nonselective cell-mediated cytotoxicity in patients with tumour progression. *Cancer Res.*, **37**, 413.

VOSE, B. M., KIMBER, I. & MOORE, M. (1977*a*) Leukocyte migration inhibition in human pulmonary neoplasia. *J. Natl. Cancer Inst.*, **58**, 483.

VOSE, B. M. & MOORE, M. (1977) Reactivity of peripheral blood leukocytes against human foetal cells. II. Cytotoxic potential of preparations enriched or depleted of different leukocytes populations. *Int. J. Cancer*, **19**, 34.

VOSE, B. M., MOORE, M. & JACK, G. D. (1975) Cell mediated cytotoxicity to human pulmonary neoplasms, *Int. J. Cancer*, **15**, 308.

VOSE, B. M., VÁNKY, F., FOPP, M. & KLEIN, E. (1978) *In vitro* generation of cytotoxicity against autologous tumour biopsy cells. *Int. J. Cancer*, **21**, 588.

VOSE, B. M., VÁNKY, F. & KLEIN, E. (1977) Lymphocyte cytotoxicity against autologous tumour biopsy cells in humans. *Int. J. Cancer*, **20**, 512.

ZINKERNAGEL, R. M. & DOHERTY, P. C. (1974) Restriction of *in vitro* T-cell-mediated cytotoxicity in lymphocytic choriomeningitis within a syngeneic or semi-allogeneic system. *Nature*, **248**, 701.